# 体外膜肺氧合期间反复动脉血栓形成1例报告及文献复习

**DOI:** 10.3760/cma.j.cn121090-20241128-00490

**Published:** 2024-12

**Authors:** 婕 靳, 静 刘, 兰娟 徐

**Affiliations:** 郑州大学附属郑州中心医院重症医学科，郑州 450000 Department of Critical Care Medicine, Zhengzhou Central Hospital Affiliated to Zhengzhou University, Zhengzhou 450000, China

## Abstract

体外膜肺氧合（Extracorporeal membrane oxygenation, ECMO）是一种基于体外循环原理，为严重心肺功能衰竭患者提供短期生命支持的先进技术，但也可出现急性肢体缺血、骨筋膜室综合征、出血和血栓等多种并发症。现回顾性分析1例58岁汉族男性急性心肌梗死合并心源性休克患者，常规治疗无效后，紧急接受静脉-动脉ECMO（VA-ECMO）治疗，在此期间反复出现动脉血栓，抗凝抗栓治疗后好转，最终因感染死亡。动静脉血栓形成导致急性肢体缺血是ECMO治疗过程中可能出现的严重并发症之一。预防血栓形成和优化抗凝抗栓方案是主要治疗措施。抗凝仍是ECMO过程中静脉血栓形成的主要治疗方法，而ECMO期间不常规抗栓治疗，但对高危动脉血栓形成风险患者抗栓为首要治疗；病情需要时早期介入或外科手术可降低缺血发生率、提高生存率，减少致残结局。

## Abstract (en)

Extracorporeal membrane oxygenation（ECMO）is an advanced technology that provides short-term life support for patients with severe cardiopulmonary failure based on the principle of cardiopulmonary bypass. However, various complications such as acute limb ischemia, osteofascial compartment syndrome, hemorrhage and thrombosis can also occur. A retrospective analysis was performed on a 58-year-old Han male patient with acute myocardial infarction complicated with cardiogenic shock in our department. After the conventional treatment failed,he received urgent VA-ECMO treatment. During this period, arterial thrombosis occurred repeatedly, improved after anticoagulant and antithrombotic treatment, and finally died due to infection. Acute limb ischemia caused by arteriovenous thrombosis is one of the possible serious complications during ECMO treatment. Prevention of thrombosis and optimization of anticoagulant and antithrombotic regimen are the main therapeutic measures. Anticoagulation is still the main treatment method for venous thrombosis during ECMO treatment. Anti-thrombotic drugs are not routinely given during ECMO, while anti-thrombotic drugs are the primary treatment for high-risk arterial thrombosis risk patients. Therefore, early detection of complications and timely intervention are crucial. Early intervention or surgical operation can reduce the incidence of ischemia,improve the survival rate and reduce the disabling outcome.

体外膜肺氧合（ECMO）是一种生命支持的手段，为危重患者提供临时心肺支持，为原发病的治疗争取更多时间，可挽救许多常规疗法难以逆转病情的危重患者。然而，ECMO的使用伴随动静脉血栓发生风险较高，Fisser等[1]对427例接受ECMO支持患者进行研究，结果显示静脉并发症发生率为27％，其中7％的患者出现肺栓塞；而动脉并发症的发生率为37％，主要表现为缺血，其次是出血、夹层和筋膜室综合征。本文我们报道了1例ECMO期间反复动脉血栓患者的诊治过程，并通过文献回顾来探讨ECMO期间反复动脉血栓的形成和防治。

## 病例资料

1例58岁汉族男性患者，身高170 cm，体重70 kg（BMI 24.2），长期抽烟30年，每日约20支；患“Ⅱ型糖尿病”30年，口服降糖药，具体用药不详，血糖未监测；冠心病、脑梗死、脑动脉支架2个月余，规律口服“阿司匹林肠溶片100 mg/d、氯吡格雷50 mg/d、阿托伐他汀钙20 mg/d”。此次因“胸闷1 h”急诊入院，心电图提示：①心房颤动伴快速心室率，平均心室率147次/min；②ST-T呈急性缺血性改变，疑急性前间壁心肌梗死；肌钙蛋白I（TnI）（−），前脑钠肽（pro-BNP）5 420 pg/ml，心脏彩超：射血分数（EF）36％（辛普森法测量），左室壁运动弥漫减低，左室收缩功能减低；血气分析：PCO_2_ 36.1 mmHg（1 mmHg=0.133 kPa），PO_2_ 63 mmHg，实际碱剩余（ABE）−6.7 mmol/L，HCO_3_^-^ 18.7 mmol/L，乳酸3.3 mmol/L。心内科综合考虑急性冠脉综合征合并急性心衰，予高流量吸氧，阿司匹林100 mg、替格瑞洛90 mg、硝酸甘油扩冠利尿30 min后胸闷症状有所缓解；99 min后胸闷加重伴烦躁，心率上升至170次/min，血压下降至92/46 mmHg，SPO_2_ 71％，全身湿冷，予血管活性药物升压、镇静、有创机械通气抢救治疗，4 min后心率44次/min，血压消失，心肺复苏25 min自主心率恢复，血压难以维持，考虑急性心肌梗死合并心源性休克，接受静脉-动脉ECMO（VA-ECMO）治疗，VA-ECMO导管在超声引导下分别置入右股静脉和右股动脉，但首次穿刺左侧股动脉失败，考虑下肢动脉硬化严重。通过超声确认导管末端在右心房水平。建立VA-ECMO后设置参数为：转速3 900/min，流量3.8 L/min，氧浓度（FiO_2_）100％，气流量4 L/min，血管活性药物：肾上腺素0.08 µg/（kg·min）和去甲肾上腺素1 µg/（kg·min）、硝酸甘油0.2 µg/（kg·min），呼吸机参数设置：氧浓度40％，呼吸频率12次/min，呼气量6 ml/kg，呼气末正压8 cmH_2_O。心肺复苏VA-ECMO时应用肝素1 000 U，术后肝素10 U/（kg·h）维持抗凝，术后4 h口腔、尿道、穿刺管路处持续出血，复查凝血指标：活化部分凝血活酶时间（APTT）>150 s，凝血与血小板功能检测（century CLOT）：凝血酶生成时间（ACT）450 s，纤维蛋白生成速率（CR）9，血小板功能（PF）1.2；血栓弹力图（TEG）：R时间18.2 min，K时间4.3 min，凝血综合指数（CI）：−11，最大振幅（MA）：63.8 mm；停用抗凝治疗，应用鱼精蛋白5 mg联合凝血酶原复合物900 U，复测ACT 220 s，APTT 80 s；每4 h监测1次ACT及APTT。当ACT<200 s或APTT<70 s开始肝素抗凝，使APTT维持在正常值1.5倍左右。

VA-ECMO上机11.5 h，床边查体发现患者双眼瞳孔散大，直径约4 mm，光反射消失，因病情不允许外出CT检查。进行多模态脑血流、灌注压、经皮脑氧、无创脑水肿及脑电图（EEG）监测，考虑缺血缺氧性脑病可能性大，冰毯亚低温、渗透治疗等集束化综合脑保护（发病48 h后瞳孔回缩，意识仍未完全恢复，颅内出血情况基本排除）。

VA-ECMO上机19.5 h，左下肢皮肤温度稍凉，且左下肢经皮血氧45％，彩超可见左侧股浅动脉远端、腘、胫前后动脉血栓形成，期间ACT及APTT均恢复目标，TEG：R 10.6 min，K 2.8 min，CI −2.9，MA 70.4 mm；新血栓四项：凝血酶-抗凝血酶复合物（TAT）26 ng/ml（正常参考范围≤4 ng/ml），纤溶酶-α2纤溶酶抑制剂复合体（PIC）3.211 µg/ml（正常参考范围≤0.8 µg/ml），均明显升高，预测血栓形成，与临床表现相符，易栓症四项：蛋白C：41％，蛋白S：21.4％，抗凝血酶Ⅲ：52％，考虑抗凝血酶Ⅲ较低，肝素敏感性因素，更换为阿加曲班0.2～0.8 mg/h抗凝，维持APTT 43.6～58.7 s。

VA-ECMO上机48 h，心脏彩超：EF 33％，左室短轴缩短率（FS）15％，二尖瓣环侧壁组织多普勒频谱（E/e′）<14，心输出量（CO）9 L/min，左室壁弥漫性运动减低，主动脉瓣、二尖瓣、三尖瓣开放尚可；前往导管室冠脉造影下见左主干、前降支、回旋支及右冠均重度狭窄，家属拒绝冠脉搭桥手术，术中应用普通肝素3 000 U，术后APTT>150 s，暂时停用抗凝药物；术后6 h APTT 45 s，再次开始阿加曲班0.4～0.8 mg/h抗凝，复查抗凝血酶Ⅲ：72％，考虑肝素仍为VA-ECMO首选，ECMO支持72 h，更换为肝素10 U/（kg·h），根据APTT调整剂量；VA-ECMO支持第4天，血压下降，脉压差小于10 mmHg，左心饱满，下调VA-ECMO流量<3 L/min，彩超可见主动脉瓣无明显开放状态，急诊介入引导下经左肱动脉成功留置左心减压主动脉球囊反搏装置（IABP）后循环状态改善；同时动态复查彩超：左侧股总动脉远心端动脉血栓、右胫前动脉远心端近闭塞，动脉血栓持续进展；PLT从189×10^9^/L降至84×10^9^/L，MA 39.8 mm；排查血小板减少原因，警惕肝素相关血小板减少（HIT），继续肝素抗凝。

VA-ECMO第5天，PT 14 s，APTT 55 s，PLT 85×10^9^/L，MA 30，输注1个治疗量血小板后，在VA-ECMO联合IABP支持下，行非体外循环下冠脉旁路移植术，手术共用6 h，术中出血1 200 ml，术后ECMO转速3 800/min，流量3.76 L/min，气流量3 L/min，肾上腺素0.01 µg/（kg·min）联合硝酸甘油1.2 µg/（kg·min），维持血压在130/55 mmHg、心率80次/min左右。术后当天未给予抗凝；术后第1天7时开始应用肝素10 U/（kg·h），APTT维持50～80 s，复查TAT：37.5 ng/ml，10时发现左下肢皮温凉，出现少量张力性水泡，左前臂皮温凉，彩超考虑左侧桡动脉闭塞，伴随左前臂出现骨筋膜室综合征表现，急诊“左上肢肱动脉切开取栓+左下肢股动脉内膜剥脱术+切开减张”，术后更换IABP至左股动脉位置，彩超见肱、尺动脉通畅，张力改善血运恢复，术后48 h，PLT 95×10^9^/L，MA 57.2 mm；联合替罗非班0.1µg/（kg·min）抗血小板聚集治疗。

术后第4天，心超：EF 39％，CO 3.5 L/min，左心室流出道速度时间积分（LVOT-VTI）15 cm，主动脉瓣开放可，心功能恢复，下调ECMO流量2.5 L/min，转速2 600/min，观察24 h，循环稳定，当日降钙素原（PCT）从1.8 ng/ml上升至12 mg/ml，WBC 22.95×10^9^/L，考虑病情重、全身多处管路，送检血及肺泡灌洗液二代测序（NGS），哌拉西林他唑巴坦升级为美罗培南1 g每8 h 1次抗感染治疗，术后第5天血和肺泡灌洗液NGS回示：碳青霉烯类耐药肺炎克雷伯菌，双下肢膝关节以下呈缺血坏死状态，双侧腹股沟区切口愈合不良，伴随渗液，应用多黏菌素B联合替加环素、利奈唑胺抗感染治疗；继续VA-ECMO流量下调至1.5 L/min后1 h循环未见波动，拟撤除VA-ECMO，但当拔除VA-ECMO动脉导管时发现股动脉无血流，考虑再发血栓形成，急诊“股动脉切开取栓”联合“导管接触尿激酶溶栓术”，继续肝素全身抗凝，间断肝素应用第9天PLT降至53×10^9^/L，血小板因子4 1.4％，4T及HEP评分均5分，考虑中高度HIT，调整为比伐卢定0.15～0.2 mg/（kg·h）抗凝，监测APTT波动50～80 s，血小板稳定恢复至正常，MA维持57.2～66.1 mm，术后第7天成功撤除IABP，序贯磺达肝葵钠7.5 mg/d+阿司匹林肠溶片0.1 g/d；实施气管切开术，发病15 d后成功脱机，进入自主呼吸康复阶段，但因下肢动脉血栓所致肢体缺血坏死、难治性多重耐药菌感染伴脓毒性休克，最终循环衰竭而死亡。

## 讨论

ECMO支持过程中的不良事件经常被报道，这可能会显著影响患者的预后。ECMO支持过程中最常见、最严重的不良事件之一是出血，其次是血栓事件和败血症。血栓发生率的报道不一致（10％～100％）[2]。ECMO期间血栓形成的危险因素很多，包括原发病打击、镇静、频繁输血、无脉搏血流、血液暴露于非生物ECMO管道等[3]，这些微环境都可能导致血栓形成，所以必须调控凝血功能。然而，ECMO期间出血的发生率高达29％，其中10％为大出血，4％～10％为颅内出血。患者可能因存在出血高危风险而抗凝不足，这也是血栓形成的重要原因。其次，在全身炎症反应的条件下，动脉损伤也是血栓形成的原因，可由解剖损伤、机械损伤、灌注不良诱发，再而急性动脉血栓形成ECMO病理生理学涉及多个相互关联的过程，包括套管管腔阻塞、管腔血栓形成的内皮损伤、血小板的持续激活以及预先存在的动脉粥样硬化闭塞性疾病、股深动脉和（或）同侧髂内动脉的意外阻塞与持续的低流量状态，许多动脉和静脉的问题发生在插管时，但也可能发生在拔管过程中[2]。本病例成人体外心肺复苏（ECPR）后，期间凝血全貌的监测及抗凝抗栓药物应用，反复出现多发动脉血栓，导致骨筋膜室综合征、肢体缺血坏死。

既往已报道文献[4]–[10]（表1）可见ECMO期间合并下腔静脉血栓形成[4]–[6]；股静脉-动脉体外膜氧合患者心室血栓形成[7],[9]–[10]；动脉血栓形成者则以主动脉根部、升主动脉[8]为主，也有左主干冠状动脉血栓形成的报道[11]，对于四肢动脉血栓反复出现者未有大量报道；2021年1篇文献报道[12]：41％的VA-ECMO患者发生导管相关深静脉血栓（Ca DVT），14％患者发生动脉并发症（急性下肢缺血多见，动静脉股瘘、晚期股动脉狭窄均不常见）；而ECMO运行期间并发感染为Ca DVT显著相关的独立危险因素，而与动脉血栓形成并无明显相关性。

**表1:** 既往文献报道静脉-动脉体外膜肺氧合（VA-ECMO）期间不同部位血栓形成及治疗结局

例号	性别	年龄（岁）	血栓部位	疾病原因	抗凝治疗	结局	参考文献
1	女	30	下腔静脉	火灾烟雾吸入心脏骤停	抗凝血栓消失	脑死亡	Chen等[4]
2	男	49	下腔静脉	冠脉动脉粥样硬化性心脏病	抗凝后血栓仍存在	生存	Chen等^4]^
3	女	25	下腔静脉	暴发性心肌炎	抗凝血栓消失	生存	付仲等^5]^
4	男	57	下腔静脉	急性ST段抬高心肌梗死	肝素抗凝	生存	Pavlov等[6]
5	女	39	下腔静脉	流行性心肌炎伴心源性休克	肝素抗凝	生存	Pavlov等[6]
6	男	63	右心室	大面积肺栓塞	肝素抗凝	死亡	Bhat等[7]
7	女	26	主动脉根部	过敏性休克	无抗凝	死亡	Hireche-Chikaoui等[8]
8	女	19	主动脉根部	恶性心律失常	肝素抗凝	生存	Hireche-Chikaoui等[8]
9	男	55	主动脉根部	冠脉三支病变	无抗凝	生存	Hireche-Chikaoui等[8]
10	男	70	左冠状动脉尖	急性心肌梗死合并心包填塞	未提及	死亡	Hireche-Chikaoui等[8]
11	男	51	升主动脉	冠脉三支病变	未提及	死亡	Hireche-Chikaoui等[8]
12	女	71	主动脉瓣尖部	ST段抬高心肌梗死	肝素抗凝	死亡	Hireche-Chikaoui等[8]
13	男	64	右心室	急性ST段抬高型心肌梗死	未提及	死亡	Alhussein等[9]
14	女	49	左心室	急性ST段抬高型心肌梗死	肝素抗凝	死亡	Alhussein等[9]
15	男	46	左心室	法洛四联征修复后严重失代偿性心力衰竭	肝素抗凝	存活	Alhussein等[9]
16-26	男9例，女2例	52.5±16.4	左心室	心源性休克	肝素抗凝	死亡	Weber等[10]

心室内血栓一旦形成，非常凶险，病死率极高，对动脉血栓形成以主动脉根部为主，股静脉-动脉ECMO时，胸主动脉和主动脉弓血流逆转，主动脉瓣血流缺失，导致主动脉内血流降低、血栓形成，在ECMO支持过程中，由于主动脉瓣关闭而导致的外周动脉搏动性丧失是一个警报信号[12]。Parzy等[13]在静脉-静脉ECMO（VV-ECMO）血栓并发症的发生率和危险因素中提到：ECMO撤除后使用CT检查Ca DVT的发生率为71.4％，ECMO期间血小板低于100×10^9^/L的比例越高，发生Ca DVT的风险越低。2022年Abruzzo等[14]在系统综述中指出，ECMO是静脉血栓栓塞症（VTE）的阳性预测因子；低回路流量、容量不足、泵速、血流速度、血流阻塞、错位、ECMO插管尺寸大、纯粹的压力以及激活血小板和凝血级联反应的多种其他因素可能导致插管相关DVT和VTE的风险增加；长期接受ECMO治疗高龄合并感染的患者在撤机后发生DVT的风险增加。Trieu等[15]研究发现，ECMO流量与体表面积（BSA）的比值（100 mm/min/m^2^）是导管相关动脉血栓形成（CaAT）的独立危险因素，每100 mm/min/m^2^ ECMO流量-BSA比值增加，发生CaAT的几率降低21％。

主要的诊断方法是心脏和下腔静脉、四肢血管的超声检查。值得注意的是，如果ECMO导管外有血栓形成，因其很容易被误认为是ECMO导管壁，所以，在应用ECMO过程中不易发现。ECMO导管拔除后，二维超声可发现管状的血栓；彩色多普勒超声显示血流可以通过血栓，甚至血栓的尾部也可以漂浮，目前还没有很好的方法鉴别这种血栓，可以通过反复测量ECMO导管壁厚来判断。因此，彩色多普勒超声可以用来对ECMO期间或ECMO导管拔除时及拔除后动脉和静脉血栓形成的检测和随访，以防止肺栓塞等严重并发症的发生。

以上患者中，大部分发现血栓后均应用肝素抗凝治疗，对于ECMO患者常规需全身肝素化，但因部分存在抗凝禁忌，采取无抗凝方案，在临床工作中，一些患者在治疗期间出现肝素诱导的血小板减少症和肝素耐药，常常需要使用替代抗凝剂；在这种情况下，直接凝血酶抑制剂（DTI），比伐卢定和阿加曲班已被确定为[16]替代品。Pollak[16]提出：与肝素相比，DTI尤其是比伐卢定对大出血事件具有更强的保护作用,对ECMO患者的临床结果有积极的影响。除了精准选择抗凝抗栓药物类型，还可选择更换导管、清除血栓、血管介入处理和手术治疗；对于四肢动脉血栓形成者，采用留置导管内溶栓药物应用也获得确切效果。文献中提到19％的患者需要进行血管手术，其中1％需进行截肢[1]。

回顾我们的患者，反复动脉血栓形成，长期糖尿病病史，虽规律药物治疗，血糖并未规律监测，糖尿病本身也会导致血管病变，该因素也因考虑在内，同时综合考虑多因素原因，完善全外显子分子遗传检测，结果显示ABCC6基因突变。大多数情况下携带ABCC6基因杂合变异个体表现出弹性纤维性假黄瘤表型的有限表现，部分患者未见相关临床表现，但皮肤超微结构表现出弹性纤维钙化，也有文献报道杂合携带者出现血管症状[17]，而该患者并无皮肤和眼部异常，而是以动脉受累为主，因此我们缺乏部分确诊该疾病的依据，患者最终死亡。我们已无法获得更多诊断依据，以后对于冠脉及四肢动静脉硬化、反复动脉血栓形成者，将详细追问病史并完善检查协助明确诊断。

根据临床病例和文献分析，我们认为反复多发动脉血栓形成导致肢体缺血坏死预后不良。血栓形成是多种因素共同作用，患者可同时存在多种易栓症危险因素，筛查时不能局限于第一发现，尤其是筛查出较脆弱危险因素而不能解释易栓症严重程度时，需进一步查找病因。预防血栓形成和优化抗凝抗栓方案是最重要一环。抗凝治疗仍是ECMO过程中血栓形成的主要治疗方法，其他外科及介入策略需要不断积累临床经验。但在现代医学中，ECMO中常见的血栓问题也一直是困扰临床医师的难题，悉尼大学医学与健康学院Goh博士团队开发了一种血栓芯片模型，利用微流体技术模拟ECMO中的血流动力学，为理解和预防血栓形成提供了新的视角[18]。同时随着人工智能技术发展，探索该技术应用于ECMO中，以实现更加精准和个性化的治疗，可预测患者对ECMO的反应和预后。
